# The Role of Autophagy Genes in Energy-Related Disorders

**DOI:** 10.3390/cells14241947

**Published:** 2025-12-08

**Authors:** Berenice Franco-Juárez, Noemí Cárdenas-Rodríguez, Luz Camacho, Saúl Gómez-Manzo, Beatriz Hernández-Ochoa, Asdrubal Aguilera-Méndez, Cindy Bandala, Luis Miguel Canseco-Ávila, Daniel Ortega-Cuellar

**Affiliations:** 1 División de Neurociencias, Departamento de Neurodesarrollo y Fisiología, Instituto de Fisiología Celular, Universidad Nacional Autónoma de México, Mexico City 04510, Mexico; bfranco@ifc.unam.mx; 2 Laboratorio de Neurociencias, Instituto Nacional de Pediatría, Secretaría de Salud, Mexico City 04530, Mexico; ncardenasr@pediatria.gob.mx; 3 Laboratorio de Nutrición Experimental, Instituto Nacional de Pediatría, Secretaría de Salud, Mexico City 04530, Mexico; lcamachoc@pediatria.gob.mx; 4 Laboratorio de Bioquímica Genética, Instituto Nacional de Pediatría, Secretaría de Salud, Mexico City 04530, Mexico; saulmanzo@ciencias.unam.mx; 5 Laboratorio de Inmunoquímica, Hospital Infantil de México Federico Gómez, Secretaría de Salud, Mexico City 06720, Mexico; beatrizhb_16@ciencias.unam.mx; 6 Instituto de Investigaciones Químico Biológicas, Universidad Michoacana de San Nicolás de Hidalgo, Morelia 58030, Mexico; amendez@umich.mx; 7 Escuela Superior de Medicina, Instituto Politécnico Nacional, Mexico City 11340, Mexico; crodriguezba@ipn.mx; 8 Laboratorio de Diagnostico y Biomedicina Molecular, Facultad de Ciencias Quimicas, Universidad Autonoma de Chiapas, Campus IV, Tapachula City 30700, Mexico; luis.canseco@unach.mx

**Keywords:** autophagy, metabolic diseases, type 2 diabetes mellitus, obesity, non-alcoholic fatty liver disease

## Abstract

Autophagy is a cellular catabolic mechanism that facilitates the degradation of cytoplasmic components, thereby restoring energy homeostasis and mitigating cellular damage. This process functions as a housekeeping system, essential for maintaining organismal viability under stressful conditions. Numerous studies have highlighted the role of autophagy in regulating various physiological processes, including metabolic pathways. Notably, certain autophagy-related genes may play a relevant role in metabolic disorders, extending beyond their involvement in the autophagic process, and may offer potential therapeutic avenues for treating energy-related metabolic diseases. This review summarizes the roles of various components of each autophagic complex and the regulators involved in the autophagic process. In particular, it explores the intricate relationship between autophagy and several metabolic diseases, including type 2 diabetes mellitus (T2DM), obesity, and non-alcoholic fatty liver disease (NAFLD).

## 1. Introduction

The triad of metabolic disorders that affect millions of individuals worldwide includes type 2 diabetes mellitus (T2DM), obesity, and non-alcoholic fatty liver disease (NAFLD). T2DM is a multifactorial condition characterized by insulin resistance and pancreatic β-cell dysfunction, resulting in the dysregulation of glucose homeostasis [1].

Obesity is a significant risk factor for the development of T2DM and contributes to NAFLD [2]. Interestingly, T2DM and NAFLD often coexist; however, NAFLD appears to be a predictor of the incidence of T2DM [3]. Recent studies suggest that autophagy plays a significant role in the regulation of glucose metabolism. Furthermore, these studies underscore the critical importance of autophagy in maintaining the homeostasis of pancreatic β-cells, as well as in other metabolic tissues that are adversely affected in T2DM [4]. Loss of autophagy genes in various tissues has been associated with hyperglycemia and insulin resistance, which are hallmarks of T2DM [5]. Additionally, dysregulation of autophagy gene expression may contribute to the pathogenesis of NAFLD [6]. Conversely, other studies suggest that the absence of specific autophagy proteins may alleviate the harmful effects associated with high-glucose and high-fat diets [7]. This review highlights the contribution of autophagy genes in the preservation of cellular and organismal functions in the context of metabolic disorders.

## 2. Overview of Macroautophagy

Macroautophagy, subsequently referred to as autophagy, is a cellular catabolic process that involves the engulfment and degradation of cytoplasmic content. This process aims to restore energy homeostasis and avoid the accumulation of damaged organelles, protein aggregates or intracellular pathogens [8]. Autophagy is mainly triggered by cellular stress conditions, such as energy exhaustion, oxidative stress, DNA damage, endoplasmic reticulum (ER) stress, mitochondrial damage, mechanical stress or pathogen infection [9]. Interestingly, studies have shown that autophagy can also be induced under conditions of nutrient abundance to prevent the release of lipids into the bloodstream by catabolism of lipid droplets (lipophagy) [10].

Yamamoto et al. described the main steps and gene products involved in every phase of autophagy, including initiation, nucleation, membrane elongation, cargo recognition, membrane closure, autolysosome formation, and recycling [11]. Briefly, in the initiation phase, the ULK complex, consisting of FIP200, ATG101, ATG13, and kinases ULK1/2, assembles in proximity to the ER membrane and adjacent to the ATG9 vesicles. This association induces the production of PIP4, facilitating the recruitment of the VPS34 kinase complex I (VPS34, Beclin-1, VPS15, NRFB2, and ATG14L), which in turn produces PI3P. This PI3P interacts with WIPI4 to anchor ATG2A, an intermembrane lipid transporter, to the phagophore. Then, ATG9A scrambles phospholipids between membrane leaflets, during the phagophore expansion [12,13,14,15,16,17]. PI3P also recruits DFCP1 (double-FYVE-containing protein 1) to form the omegasome, which is thought to serve as a scaffold for phagophore assembly [14,15,16,17].

Two conjugation systems are important for the elongation of autophagosome membrane: the ATG8 complex and the ATG12-ATG5-ATG16L1 complex [18]. The ATG12-ATG5-ATG16(L) complex is formed immediately at the beginning of autophagosome formation, where ATG12, a ubiquitin-like protein, is activated by E1-like ATG7, then transferred to the E2-like ATG10, and covalently conjugated to ATG5, then the ATG12-ATG5 complex interacts with ATG16(L) [19]. In humans, ATG8s are ubiquitin-like proteins that are subdivided into two families: LC3 (LC3A, LC3B and LC3C) and GABARAP (GABARAP, GABARAPL1 and GABARAPL2). GABARAP proteins are less studied and have no redundant effects on autophagy [18,20]. ATG8s are initially synthesized as proforms and undergo proteolytic cleavage at the C-terminus by ATG4 cysteine proteases, exposing a glycine residue. Subsequently, ATG8s are transferred to the E1-like ATG7, followed by the covalent attachment of PE by the E2-like ATG3. This process occurs in conjunction with the E3-like ATG12-ATG5-ATG16(L) complex, resulting in the production of lipidated ATG8 (PE-conjugated) [18,21,22,23].

Cargo recognition is mediated by proteins that act as selective autophagy receptors (SARs), linking ATG8 proteins to the cargo, which is commonly ubiquitinated. Among the most extensively studied receptors are p62/SQSTM1, NBR1, TAX1BP1, NDP52, CALCOCO1 and optineurin; the relevance of these receptors varies depending on the cargo targeted for degradation; however, their mechanism of action remains largely consistent [24,25]. SARs commonly feature ubiquitin-binding domains that bind with the LC3 interaction region (LIR) of ATG8 proteins. Interestingly, these cargo receptors can also interact with upstream autophagy proteins, such as FIP200, to induce the formation of an autophagosome in proximity to the cargo destined for degradation [25].

The completion of autophagosome closure involves the fusion of both autophagosome membranes and requires the participation of the Endosomal Sorting Complex Required for Transport (ESCRT) machinery, SNARE proteins, Rab GTPase, Rab-related proteins, and ATG proteins [26]. Subsequently, the fusion of autophagosomes with lysosomes is mediated mainly by SNARE proteins, leading to formation of autolysosomes. In this structure, acidic hydrolases degrade the engulfed content [27].

## 3. Role of Autophagy-Related Complexes in Metabolic Diseases

### 3.1. Initiation and Nucleation Complexes in Obesity

**Figure 1:**
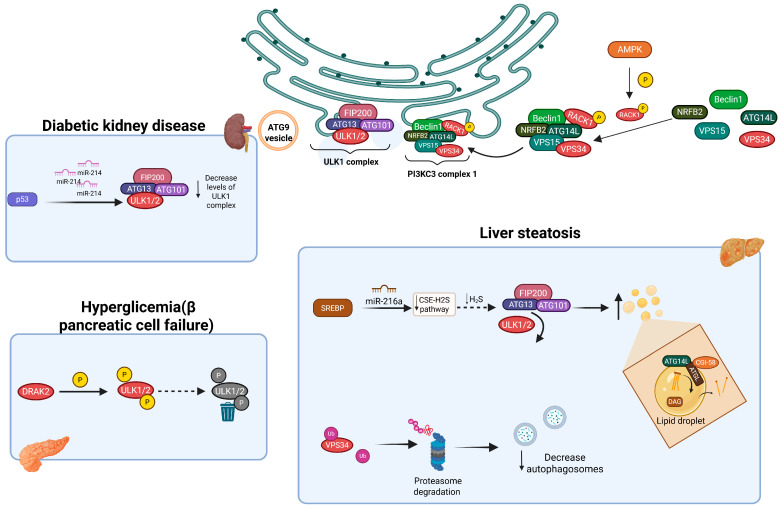
Components of the autophagy initiation complex are differentially affected across various metabolic disorders. In diabetic kidney disease, the level of ULK1/2 kinase is reduced due to the activation of miR-214, a process regulated by p53. In the context of steatotic liver, SREBP induces the expression of miRNA-216a, leading to decreased sulfhydration of ULK1 and subsequent inhibition of autophagic flux. Additionally, in pancreatic tissue affected by DMT2, the kinase DRAK2 is activated and directly phosphorylates ULK1/2, promoting their degradation and thereby suppressing autophagy. Created in BioRender. Franco, B. (2025) https://BioRender.com/i4wvc3c (accessed on 3 November 2025).

Obesity, characterized by excessive lipid accumulation predominantly in adipose tissue, is associated with a decline in overall health and has been linked to autophagy. Unc-51-like autophagy-activating kinase 1 (ULK1), a key player in the initiation complex, has been implicated in obesity pathogenesis. Nguyen et al. reported that Sterol Regulatory Element-Binding Protein (SREBP), a transcription factor that regulates genes involved in lipid synthesis, promotes the transcription of miRNA-216a, which subsequently reduces the formation of the ULK1-dependent complex. The proposed mechanism suggests that, under a high-fat diet, SREBP promotes the transcription of miRNA-216a, which then acts as a negative regulator of the Cystathionine γ-lyase (CSE)–Hydrogen Sulfide (H_2_S) pathway, a pathway that facilitates protein sulfhydration. Specifically, during high fat intake, SREBP-1c upregulates miRNA-216a, leading to downregulation of the CSE-H2S pathway and a consequent decrease in ULK1 sulfhydration (Figure 1). This reduction disrupts the interaction with ATG13 and blocks autophagic flux, ultimately contributing to lipid accumulation in the liver [28]. In contrast, liver-specific deletion of FIP200, another component of the autophagy initiation complex, confers protection against hepatic fat accumulation due to a high-fat diet, by downregulating genes involved in lipid metabolism. These findings suggest that autophagy inhibition attenuates diet-induced hepatic steatosis [29]. Together, the downregulation of distinct proteins of the initiation complex leads to different metabolic responses that might lead to lipid accumulation.

Becn1, a component of PI3KC3 complex I, has been shown to play a critical role in the pathophysiology of obesity. Specifically, deletion of Becn1 in adipocytes of murine models results in severe lipodystrophy, insulin resistance, and hepatic steatosis, accompanied by a significant reduction in survival rates attributable to fasting-induced hypothermia [30]. Furthermore, the absence of Becn1 results in decreased white adipose tissue (WAT) mass and smaller adipocyte size, likely due to enhanced adipocyte apoptosis. These findings suggest that adipocyte-specific Becn1 is an essential regulator of adipose tissue homeostasis [30,31]. Although Becn1 depletion is tissue-specific, Becn1-knockout mice exhibit lipid accumulation in the liver and metabolic alterations, including glucose and insulin intolerance [31]. Moreover, in adipose tissue, the adipocyte-specific protein PIK3C3/VPS34 is important for the differentiation, survival, and functional properties of adipocytes. Deficiency of this protein leads to dyslipidemia, characterized by abnormal blood lipid levels and the development of fatty liver disease. Additionally, deletion of Pik3c3 in murine models induces metabolic disorders that closely resemble human lipodystrophy [32].

Atg14, an additional member of PI3K complex I, has been studied in the context of liver metabolism. Specifically, Atg14 liver-specific-knockout mice subjected to a high-fat diet developed severe hepatic steatosis; however, overexpression of Atg14 in knockdown mice decreased the accumulation of lipid droplets [33]. Huang et al. also found that ATG14 localizes to lipid droplets and interacts with adipose triglyceride lipase (ATGL) and its co-activator CGI-58 to enhance the hydrolase activity of ATGL [33]. Although lipid catabolism relies on two cytosolic pathways, lipolysis and lipophagy, Huang et al. showed that ATG14 can promote triglyceride hydrolysis independently of its function in autophagy. Finally, moderate overexpression of ATG5, another component of PI3K complex I, in murine models enhances autophagy, contributes to the maintenance of leanness, and provides resistance to age-associated obesity [34].

#### 3.1.1. Initiation and Nucleation Complexes in T2DM

Similar to obesity, T2DM affects the ULK complex. Ma et al. discovered that autophagy dysfunction in diabetic kidney disease (DKD) is associated with a reduction in ULK1 levels, without alterations in factors that regulate ULK1 stability, such as FIP200, ATG13, and AMBRA1. They found that p53 mediates the induction of miR-214, which targets ULK1, leading to reduced ULK1 levels, thereby contributing to renal hypertrophy in patients with diabetes [35] (Figure 1). Another negative regulator of ULK1 was reported by Mao et al., who demonstrated that increased β-cell apoptosis induced by free fatty acids upregulates DAP-related apoptosis-inducing kinase-2 (DRAK2), a kinase that promotes ULK1 degradation [36,37]. Similarly, Lu et al. demonstrated that in pancreatic tissues from humans, macaques, and mice with T2DM, DRAK2 levels increased whereas insulin levels decreased. Interestingly, they found that DRAK2 directly phosphorylates ULK1 at serine 56, leading to its ubiquitination and degradation, which suppresses autophagy [36]. Importantly, pharmacological inhibition of DRAK2 improves β-cell function and ameliorates hyperglycemia due to autophagy promotion [36] (Figure 1). Ma et al. and Mao et al. revealed new regulators of autophagy at the induction stage, which could be targeted for therapeutic interventions to enhance autophagy. These findings highlight the role of autophagy in maintaining pancreatic β-cells and other tissues compromised during the development of T2DM.

**Figure 2:**
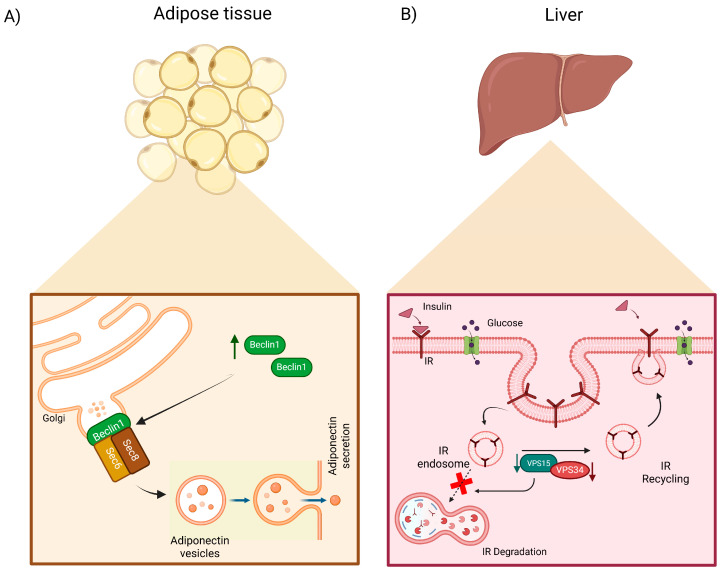
Initiation and nucleation complexes play a significant role in metabolic diseases affecting adipose tissue and the liver. (**A**) Overexpression of Becn1 in adipose tissue enhances adiponectin secretion through its interaction with Sec6, a component of the exocyst complex, thereby improving insulin sensitivity and glucose tolerance in the liver. (**B**) Reduced expression of Vps34 and Vps15 in the liver, both components of the nucleation complex, prevents insulin receptor degradation via autophagy, thereby mitigating the adverse effects associated with metabolic syndrome, T2DM, and NAFLD, including insulin resistance and glucose intolerance in several metabolic tissues. Created in BioRender. Franco, B. (2025) https://BioRender.com/tabr479 (accessed on 3 November 2025).

The induction of adipose tissue-specific expression of a mutated Becn1, which enables the constitutive activation of autophagy, improves systemic insulin sensitivity, glucose tolerance, and insulin signaling in both the liver and muscle. This enhancement occurs through the promotion of adiponectin secretion via the binding of Becn1 to Sec6, a component of the exocyst complex involved in the secretory pathway [31] (Figure 2A). Nevertheless, despite these findings, it has been proposed that T2DM augments Beclin-dependent autophagy in cardiac tissue, implying a potentially pathological role of autophagy in the heart [38].

Vps34, another component of PI3KC3 complex I, functions in conjunction with Vps15 [11,39]. Recent evidence indicates that partial inactivation of Vps34 or reduced expression of Vps15 may alleviate metabolic syndrome. Specifically, the depletion of Vps15 in the liver disrupts insulin receptor degradation, leading to enhanced insulin signaling and improved glucose metabolism [40] (Figure 2B). Furthermore, inactivation of the kinase activity of Vps34 in the liver of mice increases insulin sensitivity, reduces glucose production, and improves glucose tolerance. Interestingly, in muscle tissue, inactivation of Vps34 kinase induces a metabolic shift from oxidative phosphorylation to glycolysis, thereby enhancing glucose uptake [41]. Appropriate autophagic activity is essential for maintaining the physiological functions of renal cells. Chemical inhibition of autophagy in podocytes, achieved through the administration of 3-MA or siRNA targeting Beclin1, results in the impairment of the podocyte filtration barrier. Interestingly, a reduction in autophagy has been observed in the glomerular podocytes of mice with diabetic nephropathy, as evidenced by a significant decrease in the levels of Beclin 1, the ATG12-ATG5 conjugate, and LC3-II during the progression of diabetic nephropathy [42]. Furthermore, analyses of autophagy-related protein 5 (ATG5) and LC3B in individuals with diabetes or related pathologies, as well as in murine models of diabetic nephropathy, have demonstrated significantly reduced expression levels of these autophagy-related genes, suggesting that diminished autophagy may contribute to the development of diabetes-associated complications [43,44]

#### 3.1.2. Initiation and Nucleation Complexes in NAFLD

NAFLD is a hepatic disorder characterized by excessive fat accumulation in the liver, independent of alcohol consumption. Studies examining the effects of NAFLD in both murine models and humans with fatty liver have demonstrated that autophagic flux is inhibited due to a significant reduction in the expression of ULK1. This suppression is driven by Mir214-3p, which directly binds to the 3′ untranslated region sequence of ULK1 mRNA. These results suggest that autophagy plays a significant role in the pathogenesis of NAFLD [45].

Mice fed a high-fat diet (HFD) to induce non-alcoholic fatty liver disease (NAFLD), consistent with other metabolic disease models, demonstrated suppressed autophagy. This suppression is likely due to reduced Beclin1 activity or impaired lysosomal enzyme function. Conversely, treatment with liraglutide, an analog of glucagon-like peptide-1 that is used for the treatment of T2DM, was shown to ameliorate hepatic steatosis by enhancing autophagy [46].

One less-studied mechanism of autophagy regulation involves the degradation of core proteins. Recent studies have demonstrated that VPS34 undergoes ubiquitination, which facilitates its interaction with proteasomes and promotes its degradation. This mechanism inhibits the formation and maturation of autophagosomes, thereby negatively impacting liver metabolism [47]. Interestingly, overexpression of TRABID, an enzyme that counteracts ubiquitination, leads to the stabilization of VPS34, thereby promoting autophagy and mitigating the progression of NAFLD in a murine model [47]. In contrast, RACK1 (Receptor for Activated C Kinase 1) is involved in autophagy, particularly by facilitating the assembly of the autophagy initiation complex, which includes UVRAG, Beclin-1, Vps34, and Vps15. Mechanistically, RACK1 is phosphorylated at Thr50 by AMPK, which enhances its direct binding to Vps15, Atg14L, and Beclin-1. Consequently, RACK1 is critical for the formation of the autophagosome biogenesis complex; thus, the inhibition of RACK1 results in hepatosteatosis [41].

Moreover, impairment of autophagy in liver CD11c+ cells in mice, resulting from Atg5 deletion, promotes NAFLD through increased production of IL-23, which subsequently leads to glucose intolerance, insulin resistance, and inflammation [48]. Consistent with the role of macrophages in NAFLD pathogenesis, Kupffer cells have been identified as critical contributors. Specifically, murine models of NAFLD induced by a high-fat/fructose diet exhibit an increased abundance of Kupffer cells alongside elevated expression of DNA methyltransferases, particularly DNMT1. Activation of DNMT1 results in hypermethylation of the promoter regions of several key genes, including LC3B, ATG5, and ATG7, ultimately leading to a reduction in autophagy. Conversely, pharmacological inhibition of DNMT1 has been shown to have a beneficial effect on autophagy, thereby mitigating the progression of NAFLD [49]. In contrast, other studies have reported increased expression of autophagic conjugation genes, such as ATG5, ATG7, and ATG12, in obese individuals, suggesting elevated autophagy in the subcutaneous adipose tissue of these patients. However, these studies did not evaluate autophagic flux markers, including p62 and LC3-II, which limits the interpretation of autophagic activity [50].

### 3.2. Conjugation Complexes in Obesity

**Figure 3:**
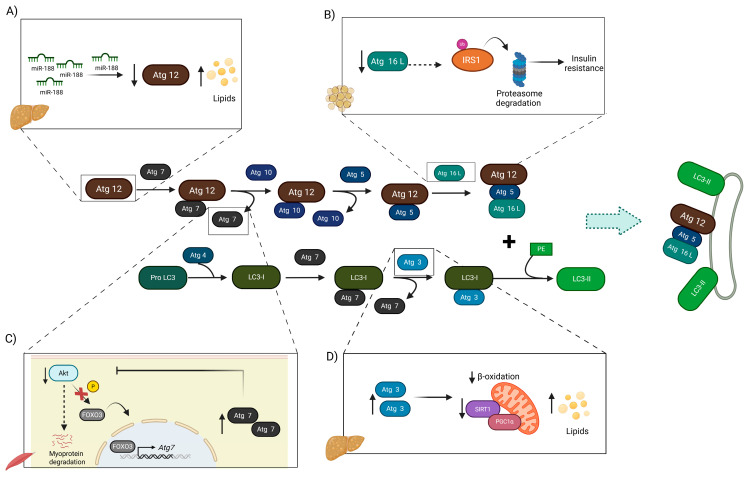
The conjugation complex components and their relationship with metabolic diseases. (**A**) ATG12 regulates lipid accumulation in mice subjected to a high-fat diet (HFD). Specifically, the HFD induces the expression of miR-188, which in turn inhibits ATG12 expression and autophagy. In contrast, activation of ATG12 ameliorates hepatosteatosis and insulin resistance. (**B**) Similarly, ATG16L positively regulates insulin sensitivity, as its deletion leads to ubiquitin-proteasome-dependent degradation. (**C**) Consistent with the deleterious effects of HFD on autophagy, this diet negatively regulates AKT signaling, thereby impairing the expression of Atg7 via its inducer FOXO3, which consequently diminishes autophagy. (**D**) Adverse effects have been attributed to ATG3, as its overexpression has been associated with decreased β-oxidation, thereby promoting lipid accumulation. Created in BioRender. Franco, B. (2025) https://BioRender.com/3t42ijj (accessed on 3 November 2025).

Research utilizing both human and animal models has demonstrated that obesity impairs muscle growth, which can be partially attributed to reductions in the size and quantity of muscle fibers. Interestingly, the expression of the E1-like protein ATG7 is elevated in the gastrocnemius muscles of obese individuals and murine models of obesity [51]. Furthermore, during obesity progression, the abundance of ATG7 in the liver increases, which may correlate with elevated serum glucose, insulin, and FFA levels. Remarkably, HepG2 and C2C12 cells treated with palmitic acid also exhibited increased ATG7 [51,52]. A high-fat diet decreases AKT activation in muscle tissue, which may subsequently lead to the activation of FOXO3. Activated FOXO3, in turn, stimulates the pathway responsible for myoprotein degradation. Interestingly, FOXO3 activation also promotes the expression of Atg7, which interacts with AKT, leading to the dysfunction of the regulatory loop that maintains muscle integrity (Figure 3C). Conversely, partial Atg7 inhibition mitigated the decline in muscle mass associated with obesity [51]. Beyond the role of ATG7 in muscle homeostasis, adipose-specific deletion of Atg7 protects mice from obesity induced by a high-fat diet and reduces hepatic fat accumulation, inflammation, and fibrosis compared to wild-type control animals [53,54,55].

ATG3 serves as a crucial component of the conjugation system, acting as an E2-like enzyme that facilitates the transfer of LC3 to PE through an intermediate LC3-ATG3 complex [56]. The deletion of ATG3 has been shown to result in defects in autophagosome formation and the degradation of the inner autophagosome membrane. However, recent evidence indicates that the inhibition of ATG3 may mitigate the onset of hepatic steatosis in the context of a high-fat diet [57,58]. In contrast, the ubiquitin-like protein ATG12 may mediate leptin sensitivity. Recent evidence indicates that mice lacking Atg12 in pro-opiomelanocortin-positive neurons (POMC) exhibit increased food intake, which subsequently leads to accelerated weight gain, heightened adiposity, and glucose intolerance when subjected to a high-fat diet [59]. Additional findings revealed that during a similar regimen of high lipid intake, ATG12 abundance was diminished due to an increased expression of miR-188 in the livers of these mice (Figure 3A). Conversely, the inhibition of miR-188 using AAV-anti-miR-188 has been shown to ameliorate hepatosteatosis and insulin resistance induced by a high-fat diet [60]. Thus, ATG12 appears to regulate lipid accumulation and the adverse metabolic effects induced by a high-fat diet. Finally, it has been demonstrated that the activation of ATG16L1-mediated autophagy through treatment with 1,25(OH)_2_D_3_ (active form of vitamin D) may decrease FFA-induced lipid accumulation and protect against hepatic steatosis [61]. Collectively, stimulation of autophagy via components of the conjugation system may help prevent lipid overload and associated alterations in insulin signaling.

#### 3.2.1. Conjugation Complexes in T2DM

Studies investigating the role of Atg7 in mice with β-cell-specific deletion of E1-like Atg7 demonstrated an accumulation of polyubiquitinated proteins. This accumulation leads to islet degeneration, characterized by a reduction in β-cell mass, impaired glucose tolerance, and decreased serum insulin levels [62]. Previous studies on the livers of ob/ob mice have reported the downregulation of autophagy markers, including a decrease in the abundance of ATG7. This downregulation is associated with impaired insulin signaling and increased ER stress [63,64]. Notably, restoration of Atg7 expression in the liver alleviates ER stress, enhances hepatic insulin action, and improves systemic glucose tolerance in obese mice. Interestingly, the depletion of ATG3 and ATG16L1 in adipose tissue has been shown to result in systemic insulin resistance and increased hepatic gluconeogenesis, both of which are characteristic features of T2DM [65]. Recent evidence indicates that hyperglycemia adversely affects the central nervous system and may contribute to neurotoxicity and neurodegeneration. This phenomenon is attributed to the S-nitrosation of ATG4B, which inhibits its activity and disrupts autophagic flux, thereby compromising the protective function of autophagy. Consequently, the inhibition of ATG4B contributes to neuronal cell death in the CNS [66]. Similarly, ATG16L, a component of the E3 complex, has been shown to regulate insulin signaling. A study conducted by Frendo-Cumbo et al. found that ATG16L1-deficient mouse embryonic fibroblasts (MEFs) exhibited insulin resistance, which was attributed to the ubiquitin-proteasome-dependent degradation of IRS1. Furthermore, mice subjected to a high-fat diet exhibited reduced levels of Atg16L1 and IRS1 in epididymal white adipose tissue, suggesting that insulin resistance may result from diminished expression of Atg16L1, leading to the subsequent degradation of IRS1 [67] (Figure 3B).

#### 3.2.2. Conjugation Complexes in NAFLD

Mutation in the autophagy-related gene ATG7 is associated with increased risk of NAFLD [68]. Patients with severe NAFLD exhibit elevated levels of a specific variant of the ATG7 gene (p. P426L variant), along with increased expression and abundance of ATG7 mRNA and protein. These findings suggest that ATG7 may play an important role in the pathogenesis of NAFLD [69,70]. Moreover, individuals diagnosed with NAFLD exhibit increased ATG3 expression, and inhibition of ATG3 may mitigate this condition. Furthermore, experiments involving human hepatic THLE2 cells treated with oleic acid indicated that silencing ATG3 in these treated THLE2 cells, as well as in mice subjected to a high-fat diet, results in a reduction in lipid storage and is associated with a significant decrease in steatosis. Notably, despite the role of ATG3 within the autophagy machinery, its overexpression can also promote lipid accumulation through autophagy-independent mechanisms by stimulating SIRT1 and CPT1a, in addition to enhancing mitochondrial function and decreasing β-oxidation [71] (Figure 3D).

## 4. Master Regulator of Autophagy: The Role of TFEB in Metabolic-Related Diseases

TFEB is a transcription factor that belongs to the MIT family of basic helix–loop–helix leucine-zipper transcription factors and is mainly recognized as the master regulator of autophagy and lysosomal biogenesis [72]. The regulation of TFEB occurs through multiple mechanisms, including transcriptional, post-transcriptional, translational, post-translational modifications, and nuclear competitive regulation. For more details, see references [73,74].

**Figure 4:**
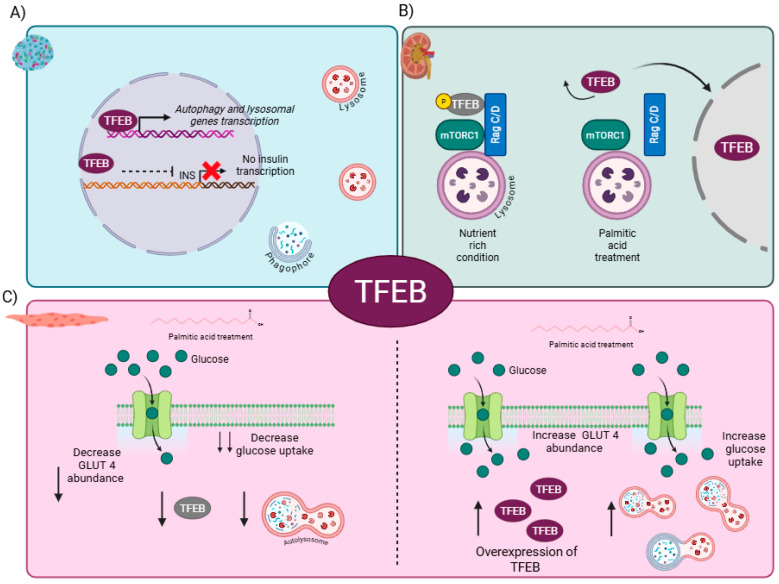
The role of TFEB in metabolic-related diseases is multifaceted. (**A**) Starvation-induced activation of TFEB, or its genetic overexpression in β cells, suppresses insulin transcription and results in glucose intolerance. (**B**) In proximal tubular epithelial cells (PTECs) cultured under nutrient-rich conditions, TFEB is phosphorylated and predominantly localized in the cytoplasm; however, in PTECs exposed to an HFD, TFEB translocates to the nucleus independently of mTORC1 signaling. (**C**) In muscle cells treated with palmitic acid, TFEB expression is diminished, resulting in impaired glucose uptake mediated by glucose transporter type 4 (GLUT4). Conversely, overexpression of TFEB restores glucose uptake and enhances autophagic flux. Created in BioRender. Franco, B. (2025) https://BioRender.com/n3ozyfy (accessed on 3 November 2025).

Recent evidence suggests that TFEB functions as a negative regulator of insulin gene expression in pancreatic β cells [75]. Starvation-induced activation of TFEB in human EndoC-βH1 cells and rat INS-1E β cells was associated with reduced insulin mRNA expression. Interestingly, gain-of-function experiments involving TFEB in both cell types showed reduced insulin expression. Furthermore, specific upregulation of TFEB in pancreatic β-cells in mice led to the suppression of insulin transcription and glucose intolerance in mice. Pasquier et al. demonstrated that TFEB binds to super-enhancer regions proximal to the insulin gene (INS) locus; however, the precise mechanism by which TFEB acts as a transcriptional repressor remains unclear (Figure 4A). It is important to emphasize that generalizations should not be made regarding the regulatory role of TFEB in different cellular contexts, including those characterized by high glucose levels or an HFD [75]. In islets from T2DM patients, TFEB was predominantly localized to the cytoplasm rather than the nucleus. This was accompanied by a reduced abundance of the lysosomal marker LAMP2, suggesting decreased autophagy. However, it is worth noting that additional markers of autophagy, such as LC3 or p62, were not assessed in this study [76].

Nakamura et al. recently proposed that in proximal tubular epithelial cells (PTECs) treated with palmitic acid bound to BSA, a model of a high-fat diet, TFEB undergoes dephosphorylation and translocates to the nucleus, thereby inducing the transcription of its target genes. This dephosphorylation event appears to be independent of mTORC1 activity, as the target proteins were unaffected by palmitic acid treatment. Instead, they were found to be dependent on reduced Rag GTPase activity [77]. Active Rag GTPase facilitates the formation of a complex between mTORC1, TFEB, and Rag GTPases, which sequesters TFEB in the cytoplasm; however, when PTECS are treated with palmitic acid, the inhibitory complex is not formed, and TFEB can migrate to the nucleus [77,78] (Figure 4B). Interestingly, the authors observed that TFEB activation peaked within the first 3 h of palmitic acid treatment, reached its maximum at 12 h, and returned to the cytoplasm after 24 h. TFEB activation in PTECs triggers lysosomal exocytosis of phospholipids as a protective mechanism against lipid overload-induced renal lipotoxicity. The relevance of TFEB in mediating lysosomal exocytosis was further supported by experiments performed in obese mice with *Tfeb*-deficient PTECs, where a high-fat diet led to the accumulation of multilamellar bodies (MBLs). Interestingly, kidney biopsies from obese patients showed decreased nuclear TFEB levels accompanied by increased vacuolation [77].

Previous research by Wang et al. has indicated that exposure of C2C12 myotubes to palmitic acid results in insulin resistance, as evidenced by reduced glucose uptake and consumption, even in the presence of insulin, along with a reduction in the abundance of GLUT4 (Figure 4C). Moreover, the study found a decline in TFEB levels following 24 h of palmitic acid treatment, consistent with the findings reported by Nakamura et al. under a similar treatment duration. Interestingly, the study found that overexpression of TFEB improve insulin resistance, furthermore they also showed that autophagy flux was restored when TFEB was overexpressed, even in the presence of palmitic acid [76] (Figure 4C).

TFEB has been recognized mainly as a lysosomal and autophagy gene regulator; however, recent evidence suggests that TFEB can influence the expression of metabolic genes such as insulin, acting as a negative regulator. Nonetheless, in other cellular contexts such as lipid overload, TFEB activation can promote phospholipid exocytosis to avoid their accumulation and can favor glucose uptake, probably by enhancing the abundance of GLUT4; however, the mechanism remains unexplored. The evidence suggests that TFEB may play an important role in metabolic diseases such as obesity and T2DM, as it promotes autophagic flux, and highlights the implications of TFEB signaling in various metabolic disorders.

## 5. Conclusions

The pathogenesis of metabolic disorders such as T2DM, obesity, and NAFLD, is complex and involves multiple cellular alterations, including those related to autophagy. Autophagy is a housekeeping cellular mechanism that maintains cellular and organismal homeostasis during stressful conditions, and failure in the activity of any component of different complexes could lead to the development of different metabolic disorders; however, in some cases, decreasing autophagy could be beneficial for the organism. The use of cellular and animal models to overexpress or lack specific autophagy genes has significantly advanced our understanding of the essential role of autophagy in preserving metabolic homeostasis. However, further investigation is required to elucidate the distinct functions of the individual autophagic components and their respective contributions to diseases associated with metabolic energy dysregulation. It is important to recognize that the overall effects of autophagy activation are context-dependent and may be dualistic; induction of autophagy can exert either protective or deleterious effects depending on the organ involved, physiological context, and extent of activation. For instance, reduced autophagy has been linked to pancreatic β-cell dysfunction, whereas inhibition of autophagy may be beneficial in muscle tissue by mitigating muscle mass loss during obesity. Thus, the net effect of autophagy appears to be tissue-specific. Moreover, given that autophagy relies on the coordinated function of interconnected autophagy complexes, elucidating the role of each component at every stage of the autophagic process may facilitate the development of targeted therapeutic strategies to counteract adverse effects associated with different metabolic disorders. Although significant challenges remain, regulation of autophagy for therapeutic purposes continues to represent a promising approach for managing diverse human diseases. Future research should aim to identify specific conditions that are most likely to benefit from clinically applicable pharmacological activators of autophagy.

## Data Availability

No new data were created or analyzed in this study. Data sharing is not applicable to this article.

## Abbreviations

The following abbreviations are used in this manuscript:

AKT	Protein kinase B
AMBRA1	Activating Molecule in Beclin-1-Regulated Autophagy 1
AMPK	AMP-activated protein kinase
ATG2	Autophagy-related protein 2
ATG3	Autophagy-related protein 3
ATG4B	Autophagy-related protein 4B
ATG5	Autophagy-related protein 5
ATG7	Autophagy-related protein 7
ATG8	Autophagy-related protein 8
ATG9	Autophagy-related protein 9
ATG10	Autophagy-related protein 10
ATG12	Autophagy-related protein 12
ATG13	Autophagy-related protein 13
Atg14	Autophagy-related protein 14
ATG14L	Autophagy-related protein 14L
ATG16(L)	Autophagy-related protein 16(L)
ATG16L1	Autophagy-related protein 16L1
ATG101	Autophagy-related protein 101
ATGL	Adipose triglyceride lipase
Becn1	Beclin-1
C2C12	myoblast cell line
CALCOCO1	Calcium-binding and coiled-coil domain-containing protein 2
CGI-58	Comparative Gene Identification-58
CNS	Central nervous system
CPT1a	Carnitine Palmitoyltransferase 1A
CSE	Cystathionine γ-lyase
DFCP1	Double-FYVE containing protein 1
DKD	Diabetic Kidney Disease
DNMT1	DNA (cytosine-5)-methyltransferase 1
DRAK2	DAP-related apoptosis-inducing kinase-2
EndoC-βH1	Human β-cell line
ER	Endoplasmic reticulum
ESCRT	Endosomal Sorting Complex Required for Transport
FFA	Free fatty acids
FIP200	FAK-family Interacting Protein of 200 kDa
FOXO3	Forkhead box O3
GABARAP	γ-aminobutyrate type A receptor-associated protein
GABARAPL1	GABA type A receptor-associated protein like 1
GABARAPL2	GABA type A receptor-associated protein like 2
GLUT4	Glucose transporter type 4
H_2_S	Hydrogen Sulfide
HepG2	human liver cancer cell line
HFD	High fat diet
IL-23	Interleukine-23
INS-1E	Insuline-1E
IRS1	Insulin Receptor Substrate 1
LAMP2	Lysosome-associated membrane protein 2
LC3A	Microtubule-associated protein 1 light chain 3 alpha
LC3B	Microtubule-associated protein 1 light chain 3B
LC3C	Microtubule-associated protein 1 light chain 3B
LIR	LC3 interaction
3-MA	3-Methyladenine
MBLs	Multilamellar bodies
MEFs	Mouse embryonic fibroblasts
mTORC1	Mammalian target of rapamycin complex 1
NAFLD	Non-alcoholic Fatty Liver Disease
NASH	Non-alcoholic steatohepatitis
NBR1	Autophagy Cargo Receptor
NDP52	Nuclear dot protein 52 kDa
p62/SQSTM1	Sequestosome-1
PE	Phosphatidylethanolamine
PI3KC3	Phosphatidylinositol 3-Kinase Catalytic Subunit Type 3
PI3P	Phosphatidylinositol-3-phosphate
PIP4	Phosphatidylinositol-4-phosphate
POMC	Pro-opiomelanocortin
PTECs	Proximal tubular epithelial cells
RACK1	Receptor for Activated C Kinase 1
SARs	Selective autophagy receptors
Sec6	Exocyst complex component Sec6
SIRT1	Sirtuin 1
SNARE	Soluble N-ethylmaleimide-sensitive fusion protein Attachment Protein Receptor
SREBP	Sterol Regulatory Element-Binding Protein
T2DM	Type 2 Diabetes Mellitus
TAX1BP1	Tax1-binding protein 1
TFEB	Transcription Factor EB
THLE2	Human liver epithelial cell line
TRABID	TRAF-binding domain protein
ULK complex	UNC-51-like kinase complex
ULK1	Unc-51–like autophagy-activating kinase 1
ULK1/2	UNC-51-like kinase 1 and 2
UVRAG	UV radiation resistance-associated gene
VPS15	Vacuolar Protein Sorting 15
VPS34	Vacuolar Protein Sorting 34
WAT	White adipose tissue
WIPI	WD-repeat protein interacting with phosphoinositides
